# THE LONG-TERM CARE STAFFING CRISIS AND COVID-19: ROLE OF THE NURSE PRACTITIONER

**DOI:** 10.1093/geroni/igac059.743

**Published:** 2022-12-20

**Authors:** Katherine McGilton

**Affiliations:** KITE Research Institute: Toronto Rehabilitation Institute-UHN, Toronto, Ontario, Canada

## Abstract

The residential long-term care sector has historically suffered from seemingly intractable staffing challenges in terms of ensuring adequate clinical expertise and a supportive work environment to address the complex health care needs of residents. Considerable evidence has demonstrated the devastating effect of COVID-19 on this fragile residential long-term care staffing structure, resulting in adverse outcomes among staff and residents alike, with the potential for permanent devastation without directed intervention. Drawing upon data from an Ontario-based study of nurse practitioner deployment during COVID-19, this talk will share an emergent approach to re-shaping expertise and capacity in Ontario, Canada through embedding nurse practitioners in residential long-term care homes. Results of this work helped to inform health policy action in the province to scale-up the use of nurse practitioners in long-term care homes, in order to enhance staff expertise and tackle the significant inequities of access to care among nursing home residents.

